# Dark-State-Mediated Photobleaching in mCherry-Based Red Fluorescent Proteins

**DOI:** 10.1021/acs.jpclett.5c04106

**Published:** 2026-03-16

**Authors:** Premashis Manna, Mark A. Hix, Srijit Mukherjee, Alice R. Walker, Ralph Jimenez

**Affiliations:** † Department of Chemistry and Biochemistry, 2647 The Ohio State University, Columbus, Ohio 43210, United States; ‡ Department of Chemistry, 2954 Wayne State University, Detroit, Michigan 48202, United States; § Department of Chemistry, 6429 Stanford University, Stanford, California 94305, United States; ∥ JILA, University of Colorado Boulder and National Institute of Standards and Technology, 440 UCB, Boulder, Colorado 80309, United States; ⊥ Department of Chemistry, 1877 University of Colorado Boulder, 215 UCB, Boulder, Colorado 80309, United States

## Abstract

Developing bright and photostable red fluorescent proteins (RFPs) is one of the “holy grails” of the protein engineering community. Despite several attempts, such fluorescent proteins (FPs) have remained elusive. One bottleneck to engineering next-generation RFPs is our lack of understanding of nonfluorescent or dark-state properties in such constructs. Here, we develop a theoretical and experimental framework that describes how photobleaching decays in FPs relate to dark-state conversion and ground-state recovery. Our systematic photophysical investigation of mCherry and mCherry-d, an RFP with enhanced dark-state behavior, showed the presence of photodestructive dark states in such FPs. Molecular dynamics simulations reveal enhanced fluctuation around the imidazolinone end of the chromophore in mCherry-d, potentially facilitating conversion to nonfluorescent states. Collectively, this work quantifies dark-state kinetics and provides insights into engineering dark states in RFPs to develop bright, yet photostable, molecular probes.

## Graphical abstract

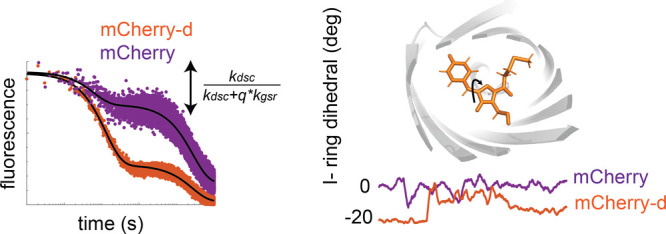

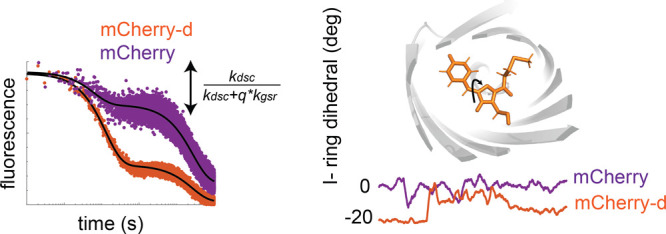

Fluorescent proteins (FPs) have gained widespread use as molecular probes in fluorescence microscopy. In the past few decades, FPs have been genetically engineered to enhance traits such as brightness, photostability, fluorescence color, and maturation time.^1^ This iterative process has yielded a diverse range of improved FPs for various applications.^2–6^ The photophysical properties of such FPs are sometimes as good as those of many small-molecule dyes. For instance, the brightness of mTurquoise2,^6^ mNeonGreen,^3^ and mScarlet3,^7^ which emit in the blue, green, and red spectral regions, respectively, is similar to that of many small-molecule dyes like Alexa, Atto, and Janelia Fluor dyes.^8^ However, the photostability of the FPs, particularly for the red fluorescent proteins (RFPs), is still far from optimal.^9,10^ For instance, tetramethylrhodamine, one of the most used small-molecule dyes in laser spectroscopy, has a *ϕ*_PB_ value of 3.3 × 10^–7^. On the contrary, the *ϕ*_PB_ value for super-photostable FPs like StayGold found in the FPbase^11^ is 3 orders of magnitude higher (*ϕ*_PB_ ∼ 10^–4^) under comparable illumination.^9,12^ This indicates that there is much room for improvement in the photostability of FPs.

It has been reported that the fluorescence brightness and photostability of FPs are inversely correlated.^13–15^ However, recent reports of bright and photostable green and yellow FPs challenge this trade-off in FP engineering.^4,12,16^ For example, StayGold, derived from the jellyfish *Cytaeis uchidae*, and its monomeric counterparts are ∼20-fold more photostable than EGFP yet maintain high molecular and cellular brightness.^12,16,17^ By employing a high-throughput single-cell screening platform, Lee et al. reported bright and photostable mGold2t and mGold2s, which are ∼25-fold more photostable than popular yellow fluorescent proteins (YFPs) such as mVenus and mCitrine.^4,18^ These improvements in photostability were not achieved at the cost of reduced brightness. However, a similar breakthrough has yet to occur in RFPs. Multiple attempts to engineer bright and photostable FPs have resulted in deterioration of one or more of a desired property for applications.^5,7,19,20^ For instance, although recent studies of mScarlet3-S2 or mScarlet3-H have reported higher photostability, these FPs sacrifice significant brightness relative to their predecessors.^21,22^

One of the main bottlenecks to improving the photostability of the RFPs while maintaining their brightness is our dearth of understanding of possible photobleaching mechanisms and how they are controlled by photoinduced nonfluorescent or dark states.^23^ Previous studies indicate that these dark states might both be photoprotective and photodestructive.^23,24^ Population transfer from the bright state to a dark state is known as dark-state conversion (DSC), whereas the relaxation from the dark state to the ground state is termed ground-state recovery (GSR) (Figure 1a).^23,25,26^ Ensemble fluorescence measurements of closely related RFPs by our lab revealed the complex kinetics of these processes over 6 orders of magnitude in time.^23^ The photobleaching kinetics of TagRFP, mKate, and several FPs from the mFruit series^27^ were fit to a triexponential function. The fast component of the decay ranging from micro- to milliseconds was attributed to DSC. On the other hand, the slower weighted biexponential component (milliseconds to seconds) was assigned to photodestruction. However, an explicit connection between microscopic parameters such as DSC, GSR, and photobleaching rates and a macroscopic observable such as photobleaching decay was not made.

**Figure 1:**
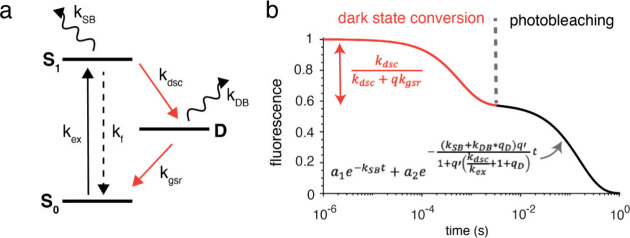
**Kinetic model for dark-state conversion and photobleaching.** (a) Three-state photophysical model of the fluorescent proteins with the ground (S_0_), excited (S_1_), and dark (D) states, along with the relevant photokinetic parameters: excitation rate (*k*_ex_), fluorescence rate (*k*_fl_), dark-state conversion rate constant (*k*_dsc_), ground-state recovery rate constant (*k*_gsr_), and photobleaching rate constants from the bright and dark states (*k*_SB_ and *k*_DB_, respectively). (b) Typical fluorescence trace obtained from the model described in panel a, where the fast decay within milliseconds (orange line) is associated with the reversible dark-state conversion and the slower decay (black line) is due to irreversible photobleaching. The relevant equations used to extract *k*_dsc_ and photobleaching decays (*k*_SB_ and *k*_DB_) are also shown in the plot. Here, $q=\frac{k_{f}}{k_{\mathrm{ex}}}+1$ , $q^{\prime }=\frac{k_{f}}{k_{\mathrm{ex}}}$ , and $q_{D}=\frac{k_{\mathrm{dsc}}}{k_{\mathrm{gsr}}}$ .

In this work, first, we derive an analytical expression that describes how the time constant and amplitude of the submillisecond fluorescence decay in FPs are governed by DSC, GSR, and excitation rates. Then, we adopt photokinetic modeling, previously used in small-molecule chromophores to reveal the interdependence of these parameters on the biexponential photobleaching observed in FPs.^28–30^ Second, we employ this method to extract the kinetic parameters of mCherry^27^ and mCherry-d, an RFP that we selected using a lifetime and photostability-gated microfluidic cell sorting system developed by Dean et al.^15^ mCherry and mCherry-d are closely related and have similar molecular brightness (i.e., extinction coefficient × fluorescence quantum yield) yet display contrasting dark-state and photobleaching properties upon illumination in the kilowatt per square centimeter regime (1–10 kW/cm^2^). This intensity regime is relevant for typical confocal laser scanning microscopies. Our analysis indicates a photodestructive dark state in mCherry-d. Intensity-dependent photobleaching measurements of the RFPs reveal that a higher illumination intensity amplifies both the kinetics and the amplitude of dark-state-mediated photobleaching. Finally, to investigate the molecular origin of such dark states, we performed all-atom explicit solvent molecular dynamics (MD) simulations. We discovered a significant destabilization of the phenolate ring (P-ring) and imidazolinone ring (I-ring) of the mCherry-d chromophore relative to its precursor, mCherry. This is particularly noteworthy for the I-ring of the chromophore, as revealed by a large deviation of the I-ring dihedral angle from zero, as well as enhanced fluctuations. We present analytical formulas for quantifying dark-state properties from time-resolved fluorescence measurements, which are pivotal for designing high-throughput selection strategies for improved photophysical parameters. Identification of the molecular origin of dark states will guide the design of FP libraries to stabilize or eliminate these states for the application of localization-based super-resolution microscopies or wide-field/confocal imaging, respectively.^31^

We describe the photokinetics of the RFPs by a three-state photophysical model, as described in Figure 1a. Upon illumination, the chromophore of the RFP is excited from the ground state (S_0_) to the excited state (S_1_). From S_1_, the molecule can be trapped in a dark state (D) and subsequently relax back to S_0_. The rate constants for entering and exiting D are *k*_dsc_ and *k*_gsr_, respectively. The model assumes that photobleaching occurs from either the singlet excited state or the dark state with a characteristic rate constant of *k*_SB_ or *k*_DB_, respectively. Here, the time constant of any process is taken as the reciprocal of the relevant rate constant (i.e., *τ*_dsc_ = 1/*k*_dsc_).

The typical rate constants for excitation (*k*_ex_) and fluorescence (*k*_f_) are on the order of a few megahertz (for irradiance regimes nearing optical saturation, i.e., ∼kW/cm^2^ intensity) and hundreds of megahertz (∼ns), respectively (see section S1 of the Supporting Information). On the other hand, the photobleaching, DSC, and GSR processes are significantly slower, in the range of a few kilohertz (approximately milliseconds) to tens of kilohertz (50 μs).^23,25,26^ These slower kinetics are consistent with the assumption that the dark and bleached states are effectively nonabsorptive to the excitation photons driving the S_0_–S_1_ transition. Therefore, the kinetics of the S_0_ and S_1_ states can be separated from the dark-state conversion or photobleaching processes. In this limit, a rapid equilibrium is assumed between the S_0_ and S_1_ states, i.e., *k*_f_[S_1_]_0_ = *k*_ex_[S_0_]_0_. Here, [S_0_]_0_ and [S_1_]_0_ are the equilibrium populations of the corresponding states. The validity of this approximation is justified by numerically solving for the exact populations in the ground and excited states (section S1).

At the illumination intensities employed here (1-10 kW/cm^2^, typical for confocal imaging), the characteristic dark-state conversion (DSC) time constant (tens of microseconds) can be an order of magnitude shorter than that of irreversible photobleaching, which typically occurs on time scales of tens of milliseconds. Consequently, over short observation windows (<1 ms), photobleaching can be treated as negligible. This approximation led us to derive an analytical expression of the excited-state population

1 $${\left[S_{1}\right]}_{\mathrm{norm}}=\frac{k_{\mathrm{dsc}}}{k_{\mathrm{dsc}}+qk_{\mathrm{gsr}}}e^{-\left(k_{\mathrm{gsr}}+\frac{k_{\mathrm{dsc}}}{q}\right)t}+\frac{qk_{\mathrm{gsr}}}{k_{\mathrm{dsc}}+qk_{\mathrm{gsr}}}$$

2 $$a_{\mathrm{dsc}}=\frac{k_{\mathrm{dsc}}}{k_{\mathrm{dsc}}+qk_{\mathrm{gsr}}}$$

where $q=\frac{k_{f}}{k_{\mathrm{ex}}}+1$ and *a*_dsc_ is the DSC amplitude. Detailed derivation of these equations and their validation from numerical simulation are described in section S2.

Figure 1b shows a representative fluorescence bleaching trace simulated by using the three-state model described in Figure 1a. The corresponding rate equations were solved numerically, and the normalized excited-state population (S_1_) was used as a proxy for the fluorescence signal. When plotted on a logarithmic time axis, the fluorescence trace exhibits an initial rapid decay on the submillisecond to ∼1 ms time scale, followed by a quasi-steady plateau and a subsequent slow exponential decay spanning the millisecond to second regime. The early time decay arises predominantly from dark-state conversion (orange trace in Figure 1b), with a negligible contribution from irreversible photobleaching over this interval. In contrast, the long time decay beyond ∼1 ms is dominated by irreversible photobleaching processes (black trace in Figure 1b).

As indicated by eq 1, at short time scales (within 1 ms) where photobleaching is negligible, the fluorescence decay is described well by a single-exponential function. Given that either *τ*_gsr_ or *τ*_dsc_ is known from independent time-domain experiments,^25^ the remaining parameter can be extracted from fits to the experimental fluorescence decay (Figure 2). In this work, the *τ*_gsr_ for the RFPs was determined from independent time-domain measurements (Figure 2a,b) and subsequently used to extract *τ*_dsc_. We termed the amplitude of this fast decay the “DSC amplitude” (*a*_dsc_, eq 2). In section S3, we show how *a*_dsc_ depends on various other kinetic parameters of our three-state model. In theory, both the DSC amplitude ( $\frac{k_{\mathrm{dsc}}}{k_{\mathrm{dsc}}+qk_{\mathrm{gsr}}}$ ) and the decay exponent ( $k_{\mathrm{gsr}}+\frac{k_{\mathrm{dsc}}}{q}$ ) can be used to determine DSC time constants. In practice, however, the decay exponent is highly sensitive to a small number of early time data points and is therefore prone to sampling errors. By contrast, *a*_dsc_ can be extracted more robustly, providing a reliable measure of DSC time constants over a broader parameter range, as demonstrated in section S3 (Figure S3.1).

**Figure 2:**
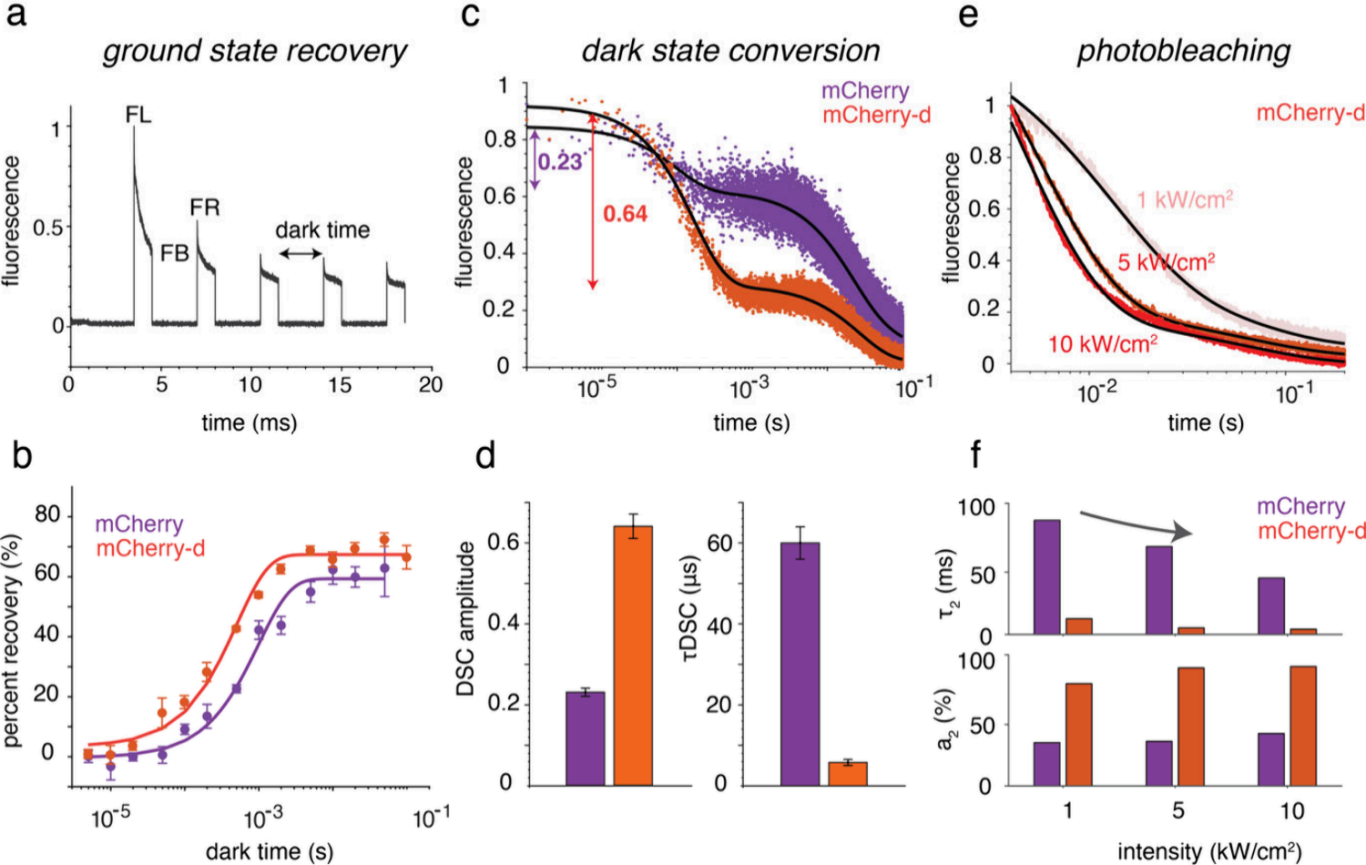
**Dark-state and photobleaching properties of mCherry and mCherry-d.** (a) Typical fluorescence signal obtained from an FP sample in a ground-state recovery (GSR) experiment. Cells expressing RFPs are excited with a 2 ms pulse and varying interpulse delays or dark times. Values of FL, FB, and FR are extracted from these experiments to calculate percent recovery employing eq 4. (b) Percent recovery vs dark time (dots) measured at 10 kW/cm^2^ fit to a single exponential (solid lines) to obtain GSR time constants. The error bars are standard deviations from three technical replicates. (c) Fluorescence traces demonstrating higher-amplitude dark-state conversion in mCherry-d (orange) compared to mCherry (purple) at 5 kW/cm^2^. While the fraction of fluorescence decrease within 1 ms for mCherry is only 0.23, it is 0.64 for mCherry-d. The black lines are exponential fits. (d) DSC amplitude and time constants (*τ*_DSC_) for mCherry and mCherry-d shown as purple and orange bars, respectively. The error bars are standard deviations from at least four independent measurements. (e) Normalized photobleaching decays of mCherry-d under continuous illumination at 1, 5, and 10 kW/cm^2^. For each sample, the decays at different intensities are globally fit with a biexponential function (black lines). In the fit, τ_1_ is kept as a shared variable whereas the amplitudes (*a*_1_ and *a*_2_) and τ_2_ are intensity data specific. (f) Amplitudes and time constants of the second component of mCherry (purple) and mCherry-d (orange) at different intensities.

It is noteworthy that although eq 1 can be used to estimate both *τ*_gsr_ and *τ*_dsc_ from experimental fluorescence traces by fitting, such analyses generally incur substantial uncertainty. Consequently, independent measurements are required to reliably quantify these rates. For instance, in a previous report,^25^ we obtained GSR rates from time-domain measurements and then utilized these rates to extract *τ*_dsc_ in a phase-based frequency-domain measurement. In a separate report, *τ*_gsr_ values were determined from off times of single-molecule blinking in RFPs using TIRF-based measurements, while *τ*_dsc_ was extracted by solving the corresponding eigenvalue equations and fitting the resulting model to the ensemble fluorescence decay in bacteria.^26^ In both cases, however, an explicit dependence of the dark-state kinetics on the fluorescence decay was not considered. This omission is justified, for example, in the case in which the irradiation levels in single-molecule experiments (∼ W/cm^2^) are approximately 3 orders of magnitude lower than those used here, resulting in excitation rates that are not rate-limiting. Under these conditions, the dominant time scale reflects depopulation of the dark states, manifested primarily as off times in single-molecule trajectories corresponding to *τ*_gsr_. In contrast, the present work explicitly reveals and quantifies the dependence of fluorescence decay on dark-state kinetics under high-irradiance conditions, typical for confocal imaging.

Now, we adopt an analytical expression for photobleaching decays as solved for small-molecule dyes with a similar three-state form (Figure 1a).^30^ In this case, by applying the rapid equilibrium approach as discussed above, the fluorescence decay (*F*(*t*)) can be expressed as

3 $$F\left(t\right)=a_{1}e^{-k_{\mathrm{SB}}t}+a_{2}e^{\left(k_{\mathrm{SB}}+k_{\mathrm{DB}}q_{D}\right)q^{\prime }/1+q^{\prime }\left(\frac{k_{\mathrm{dsc}}}{k_{\mathrm{ex}}}+1+q_{D}\right)t}$$

where *a*_1_ and *a*_2_ are constants, $q^{\prime }=\frac{k_{f}}{k_{\mathrm{ex}}}$ , and $q_{D}=\frac{k_{\mathrm{dsc}}}{k_{\mathrm{gsr}}}$ . The details of the derivation are presented in section S4. As one can clearly see from eq 3, the photobleaching decays are biexponential, in agreement with our experimental results. The first term is due to the decays from solely the S_1_ state (i.e., *k*_SB_), while the second term involves photobleaching rate constants from the S_1_ and D states (i.e., *k*_SB_ and *k*_DB_, respectively). The second component also depends on the excitation rate (*k*_ex_), DSC rate constants (*k*_dsc_), and the ratio of DSC and GSR rate constants (*q*_D_).

Next, we apply the theoretical formulation developed above to the experimental data to extract relevant kinetic parameters. For this, we performed systematic photophysical characterization of mCherry and mCherry-d (“d” for dark), a mutant of mCherry characterized by a higher amplitude of dark-state formation. The mCherry-d mutant was developed via directed evolution of mCherry using a high-throughput microfluidic sorter as described in section S5 and ref 32. Relative to mCherry, this variant contains three internal mutations (I161M, Q163M, and I197R) near the phenolate end of the chromophore. Also, it contains the K70R internal mutation located below the methine bridge of the chromophore connecting the phenolate and imidazolinone moieties of the MYG chromophore, which is typical for several RFPs. These amino acid positions are reported to play important roles in brightness, maturation, and photostability in FPs.^2,27,33^ The full set of mutations in mCherry-d is provided in Table S1.

The photophysical parameters of mCherry and mCherry-d are listed in Table 1. The blue-shift of the absorbance maximum is consistent with the incorporation of the positively charged I197R mutation, as reported previously.^34^ MD simulations suggest the incorporation of the I197R mutation into mCherry-XL leads to the formation of multiple H-bonds, which enhances the overall rigidity of the chromophore.^20,34^ The reduction of chromophore flexibility is consistent with the longer excited-state lifetime in mCherry-d compared with that of its precursor. Similar enhancements of fluorescence have been demonstrated for rigidified chromophores in other FPs, even with a nonplanar chromophore.^35^ Although the excited-state lifetime of mCherry-d is ∼30% longer than that of mCherry, they have very similar fluorescence quantum yields. Detailed photophysical characterizations, including the measurements of excitation and emission spectra, excited-state lifetimes, quantum yields, and extinction coefficients, are given in section S6.

**Table 1:** Photophysical Properties of mCherry and mCherry-d Variants

RFP	*λ*_abs_ (nm)	*λ*_ems_ (nm)	τ (ns)	ϵ (M^–1^ cm^–1^)	ϕ	*τ*_dsc_ (μs)^b^,^c^	*τ*_gsr_ (ms)^c^,^a^
mCherry	587	610	1.72	74,000	0.25	60 (5.7)	1.0 (0.9–1.6)
mCherry-d	572	608	2.22	55,000	0.27	5.8 (0.8)	0.5 (0.3–0.7)

a Values in parentheses are standard deviations from three to five biological replicates.

b *τ*_dsc_ and *τ*_gsr_ are intrinsic parameters; i.e., they are not dependent on excitation intensity.

c Values in parentheses are 95% confidence intervals.

Under the experimental scheme presented in panels a and b of Figure 2, yeast cells expressing mCherry and mCherry-d are excited with a pulse with an exposure time of 2 ms and varying interpulse delays (dark time) ranging from 5 μs to 100 ms in a custom-built inverted microscope.^25^ A 532 nm laser was used to irradiate the sample at a rate of 10 kW/cm^2^ (intensity measured at the sample plane). Figure 2a shows a typical fluorescence trace obtained from such an excitation. Fluorescence intensities at points FL, FB, and FR in Figure 2a are extracted from such experiments to calculate the percent recovery (PR) employing

4 $$\mathrm{PR}=\frac{\mathrm{FR}-\mathrm{FB}}{\mathrm{FL}-\mathrm{FB}}\times 100$$

The PR values obtained from eq 4 were plotted as a function of the dark time and fitted to a single-exponential function to extract the GSR time constants (Figure 2b). Using this analysis, we found that mCherry-d exhibits approximately 2-fold faster recovery kinetics compared to mCherry, with time constants of 0.5 and 1 ms, respectively, suggesting that the depopulation from the dark state into the ground state is faster in mCherry-d. Additional details of the GSR measurements and analysis are provided in section S7.

We next focus on DSC kinetics. For the measurements of DSC time constants, the fluorescent proteins expressed in yeast cells were irradiated with a 561 nm laser at 5 kW/cm^2^ (intensity measured at the sample plane) in a setup similar to that presented previously.^25^ Panels c and d of Figure 2 demonstrate the starkly different dark-state conversion in mCherry and mCherry-d. To extract the DSC amplitude and time constants, the normalized fluorescence decays from yeast cells are fit to a triexponential function. Although the DSC decay can be fit to a single-exponential function at short times (eq 1), the presence of photobleaching components in observed fluorescence requires a triexponential fit. However, the faster microsecond component and the corresponding amplitude from these triexponential fits are taken as the DSC exponent and DSC amplitude, respectively. As discussed above, the DSC amplitude is a better quantity to extract *τ*_dsc_ compared with the exponent. Therefore, DSC rate constants are obtained using eq 2. *τ*_gsr_,*k_ex_* and *k_em_* are measured independently. The details of these measurements and analysis are given in section S8.

We measure the DSC time constant of mCherry as 60 μs, which is ∼10-fold-slower than that of mCherry-d (*τ*_DSC_, 6 μs). This difference is evident from the pronounced fluorescence decrease within the first millisecond of illumination for mCherry-d compared to that for mCherry (Figure 2c). Moreover, the DSC amplitude increases from 0.23 in mCherry to 0.64 in mCherry-d, suggesting the stronger propensity of mCherry-d to populate dark states.^23^ In our model, although the amplitude of DSC (*a*_dsc_) is dependent on intensity (eq 2), the corresponding time constant (*τ*_dsc_) is treated as an intrinsic property of the fluorescent proteins. Similarly, *τ*_gsr_ was modeled as an intrinsic property of the sample. Consistent with this assumption, measurements of GSR time constants for RFPs acquired at different excitation intensities did not show any significant variation.^25^

Figure 2e shows the normalized photobleaching decay of mCherry-d expressed in yeast cells at 1, 5, and 10 kW/cm^2^ under continuous illumination. For each sample and intensity, three independent measurements were taken. The initial submillisecond fluorescence decay is largely due to dark-state conversion and therefore excluded from irreversible photobleaching analyses and fits. For each sample, the remaining background-corrected decays at different intensities are globally fit with a biexponential function of the form

5 $$a_{1}e^{-t/\tau_{1}}+a_{2}e^{-t/\tau_{2}}+b$$

where *a*_1_ and *a*_2_ are the amplitudes of the decay, τ_1_ and τ_2_ are photobleaching time constants, and *b* is a constant. As shown in eq 3, the photobleaching decay can be modeled as biexponential where the first term depends only on photobleaching from the S_1_ state (i.e., *k*_SB_), while the second exponent depends on *k*_ex_, which is related to laser intensity. Therefore, for each sample, decays with different intensities are globally fit with a biexponential function where τ_1_ is kept as a shared variable. On the other hand, the amplitudes (*a*_1_ and *a*_2_) and τ_2_ are kept as dependent on intensity data. The fits are shown as black lines in Figure 2e. Here, τ_1_ is the reciprocal of *k*_S_1_B_ and therefore is an intrinsic time constant whereas τ_2_ is an intensity-dependent parameter.

Table 2 displays the decay constants and amplitudes obtained from such global fits under continuous illumination. As expected, within each sample, the weighted bleaching time constants (*τ*_avg_) become faster with an increase in illumination intensity. However, at the same intensity, mCherry-d bleaches ∼3-fold faster than mCherry. For instance, at 5 kW/cm^2^, *τ*_avg_ for mCherry is 29 ms whereas it is 11 ms for mCherry-d. The first bleaching time constant (τ_1_), which is essentially the reciprocal of *k*_SB_, is slower in mCherry-d (10 ms vs 63 ms). However, our analyses suggest that the higher amplitude and faster time constants of the second component (*a*_2_ and τ_2_, respectively) largely contribute to the enhanced photobleaching in mCherry-d.

**Table 2:** Photobleaching Time Constants of mCherry and mCherry-d under Continuous Illumination Obtained from Globally Fitting the Decays at Three Different Intensities^a^

	intensity (kW/cm^2^)	*a*_1_	τ_1_ (ms)	*a*_2_	τ_2_ (ms)	*τ*_avg_ (ms)
mCherry	1	67 (0.1)	10 (0.03)	33 (0.2)	87 (0.2)	35
	5	66 (0.1)		34 (0.2)	67 (0.2)	29
	10	60 (0.1)		40 (0.2)	43 (0.1)	23
mCherry-d	1	22 (0.2)	63 (0.1)	78 (0.1)	12 (0.03)	23
	5	10 (0.3)		90 (0.04)	5 (0.01)	11
	10	9 (0.5)		91 (0.04)	4 (0.01)	9

a *τ*_1_ is a shared variable (intrinsic time constant), and *τ*_2_ is data specific (intensity-dependent). Values in parentheses are the 95% confidence intervals of the fitted parameters.

Figure 2f plots *a*_2_ and τ_2_ at different intensities for mCherry and mCherry-d. For both FPs, we find that the amplitude of *a*_2_ increases and the corresponding decay constants (τ_2_) become faster with an increase in laser intensity. More interestingly, at the same intensity, *a*_2_ for mCherry-d is significantly higher and τ_2_ is faster than that of mCherry. For instance, at 5 kW/cm^2^, *a*_2_ values for mCherry and mCherry-d are 34% and 90%, respectively. On the other hand, τ_2_ is ∼13-fold faster for mCherry-d (67 ms vs 5 ms), resulting in a markedly faster overall decay. As the second component involves DSC and GSR rates (eq 3), these results indicate that the accelerated photobleaching decay in mCherry-d is mediated by dark-state dynamics.

To further investigate the dark-state-mediated photobleaching in these RFPs, we compared their fluorescence decays under continuous and pulsed illumination at 1, 5, and 10 kW/cm^2^. For pulsed excitation, a Gaussian-shaped beam with a full width at half-maximum (fwhm) of 0.2 ms and an interpulse delay of 0.65 ms was used. This temporal profile was chosen to mimic the excitation conditions experienced by cells in the microfluidic flow cytometer (section S6), conditions that are relevant to the screening and selection of mCherry-d. Panels a and b of Figure 3 show the photobleaching decays (black lines) of mCherry and mCherry-d under pulsed excitation, respectively. The decays were locally fit to a biexponential function (eq 5), with the fits and parameters presented in Figure S10 and Table S3. Figure 3c summarizes the average photobleaching time constants (*τ*_avg_) under both illumination modes. At the same average intensity, pulsed illumination substantially reduces the extent of photobleaching compared to continuous illumination. For example, at 5 kW/cm^2^, mCherry photobleaches 3 times slower under pulsed excitation (91 ms) than under continuous illumination (29 ms) while mCherry-d exhibits a 5-fold increase in photostability (50 ms vs 11 ms). This enhancement is consistent with the involvement of photodestructive dark states. As pulsed excitation allows population recovery from these dark states, photostability is enhanced. Additionally, the faster ground-state recovery (GSR) of mCherry-d compared to that of mCherry (0.5 ms vs 1 ms) contributes to its improved relative performance (5-fold in mCherry-d vs 3-fold in mCherry) under pulsed excitation.

**Figure 3:**
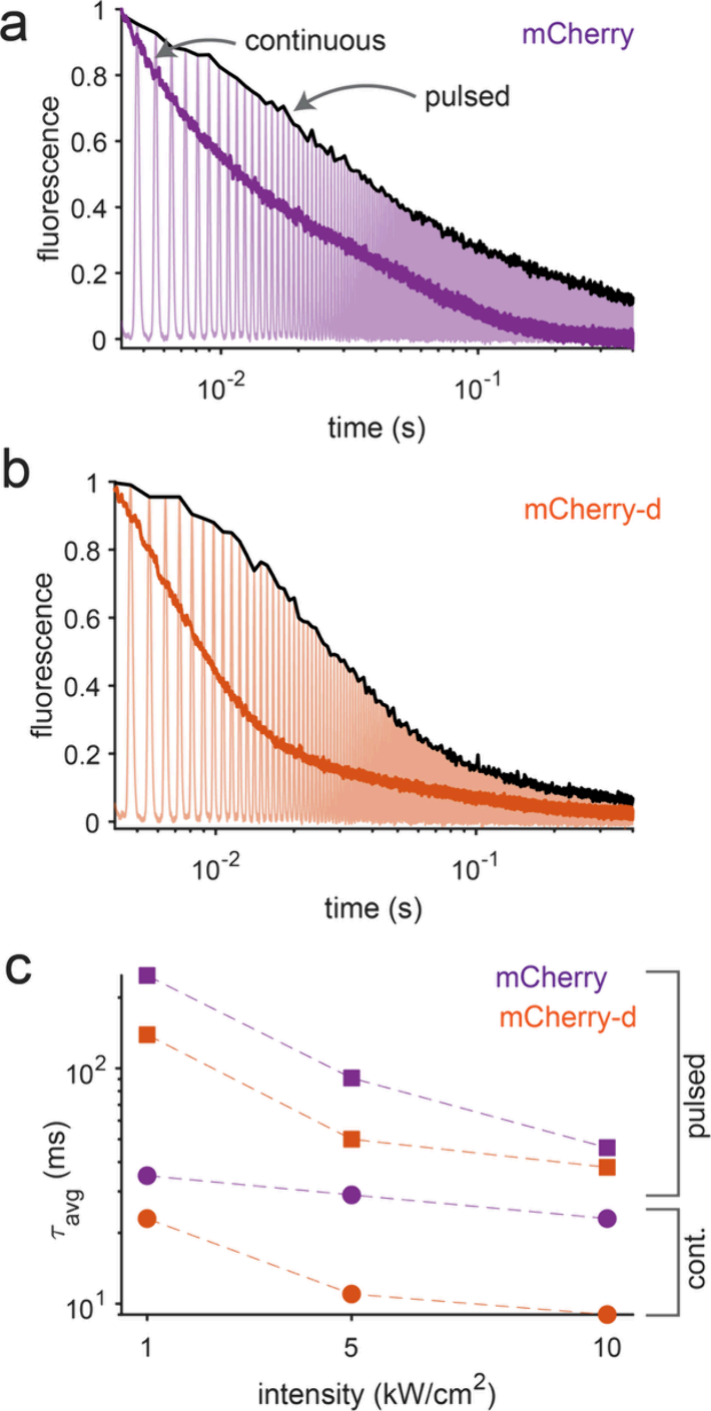
**Photobleaching decays under pulsed illumination**. Representative photobleaching decays at an average illumination intensity of 5 kW/cm^2^ (measured at the sample focal plane) were observed for (a) mCherry and (b) mCherry-d. The normalized continuous photobleaching decays are shown in dark purple and dark orange for mCherry and mCherry-d, respectively. The normalized fluorescence traces obtained under pulsed illumination conditions are shown in light purple and light orange for mCherry and mCherry-d, respectively. In the pulsed mode, a Gaussian-shaped excitation beam with a fwhm of 0.2 ms and an interpulse delay of 0.65 ms was used. The peaks of the fluorescence measured under this condition are connected to generate the pulsed photobleaching decays (black lines). (c) Average photobleaching decays of mCherry (purple) and mCherry-d (orange) under continuous (filled circles) and pulsed (filled squares) illumination conditions.

Together, these kinetic analyses suggest that the enhanced accumulation of dark-state populations in mCherry-d renders it more susceptible to irreversible photobleaching originating from these states, which are therefore photodestructive. This is consistent with our previous report in which we showed faster photobleaching in mCherry under continuous illumination compared to a pulsed illumination, indicating a photodestructive dark state.^23^ On the contrary, several eqFP578-based RFPs such as TagRFP^23^ and FusionRed^24^ mutants are reported to have photoprotective dark states, albeit at significantly lower illumination intensities. Nevertheless, the comparative photobleaching studies presented above indicate that FPs possessing photodestructive dark states, such as mCherry, are expected to exhibit improved photostability under pulsed illumination. Additionally, lower excitation intensities and FPs with faster ground-state recovery reduce population trapping in dark states, thereby mitigating irreversible photobleaching.^36^

The discovery of photodestructive dark states in mCherry mutants motivated us to investigate their possible molecular origin. Although such photophysics is inherently governed by the excited-state manifold of the FPs, the conformational heterogeneity in the electronic ground state revealed by molecular dynamics simulations can provide valuable mechanistic insights. Therefore, we performed all-atom MD simulations with AMBER and explicit solvent (TIP3P) on mCherry and mCherry-d starting with the reported high-resolution crystal structure of mCherry (PDB entry 2H5Q). Further simulation details are presented in section S11. The simulations suggest that the Q163M substitution in mCherry-d disrupts the hydrogen bonding (H-bonding) network at the phenolate end of the chromophore, while the K70R and I197R mutations alter the H-bonding network around the imidazolinone end of the chromophore. For instance, in mCherry-d, the chromophore has more hydrogen bonding with R70 (+36% vs 70K in mCherry) but less hydrogen bonding with M163 (−23% vs 163Q in mCherry). In addition, R197 in mCherry-d forms more hydrogen bonds with the β-barrel backbone compared to I197 in mCherry, (e.g., a triad with K/R70 and E148) displacing and stabilizing the chromophore, despite not directly hydrogen bonding with it. These residues are highlighted in Figure 4a, and the associated disruption of the H-bonding network is demonstrated in Figure S11. Collectively, these mutations reposition the chromophore toward R70 and promote altered dihedral twisting. Similar trends were observed in our previous systematic study that led to the development of mCherry-XL, the brightest mCherry mutant reported to date, in which decreased chromophore and protein flexibility correlated with enhanced fluorescence brightness along a fluorescence–lifetime trajectory.

**Figure 4:**
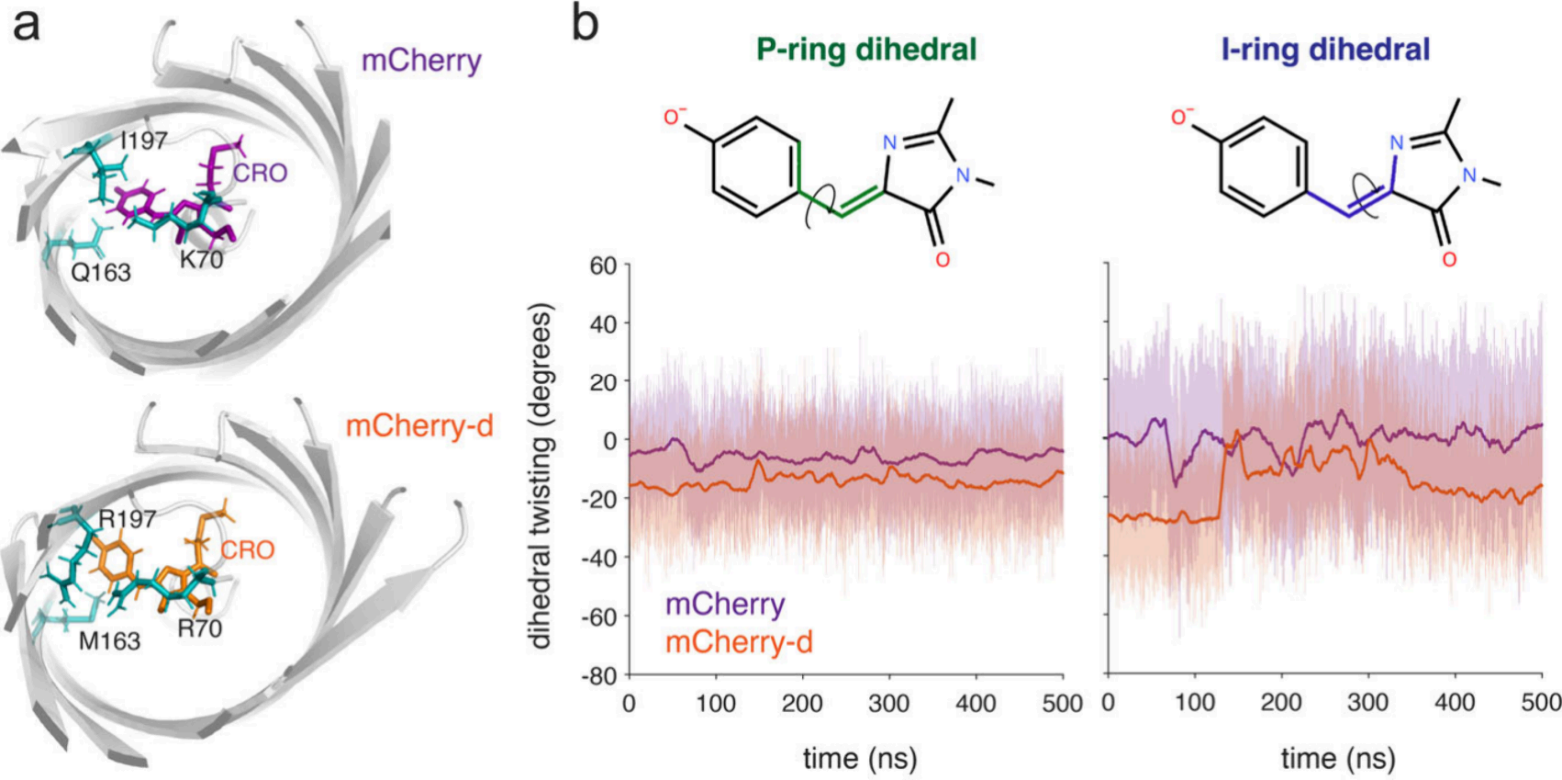
**Proposed molecular origin of dark states in mCherry-d.** (a) Key residues responsible for the disruption of hydrogen bond networks in mCherry (top) and mCherry-d (bottom) in an mCherry crystal structure (PDB entry 2H5Q). The mutations in mCherry-d are introduced in silico. The chromophores (CRO) are shown in purple for mCherry and orange for mCherry-d. The FP barrel structures are colored gray. (b) Phenolate-ring (left) and imidazolinone-ring (right) dihedral twisting as a function of time for mCherry (purple) and mCherry-d (orange) as obtained from MD simulations. The solid lines are the moving averages of the traces. The dihedral angles quantified are shown for each plot.

Altered hydrogen bonding at the phenolate end also affects the dihedral angles along the chromophore bridge. An increased degree of rotational freedom facilitates access to nonradiative decay pathways in the excited state, which is reflected in enhanced ground-state twisting and reduced chromophore planarity,^37,38^ features experimentally associated with lower fluorescence quantum yields and theoretically with increased accessibility to conical intersections for the chromophore.^20,38^ Figure 4b displays the P- and I-ring dihedrals for mCherry (purple) and mCherry-d (orange) within 500 ns. Wild-type mCherry demonstrates I- and P-ring dihedral twisting from −20° to 20°, centered at 0°. mCherry-d, however, has P-ring rotations from −40° to 20° centered at −18°. We correlate this to the Q163M mutation and the loss of hydrogen bonding at the phenolate end of the chromophore. Previously, Regmi and co-workers used fixed-charge, explicit solvent all-atom MD simulations to show that the Q163M mutation alters the oxygen diffusion channels in mCherry.^39,40^ This resulted in a significantly higher rate and a significantly higher number of oxygen molecules entering the protein barrel compared to those of wild-type mCherry, contributing to enhanced photobleaching. Similar oxygen-dependent photobleaching may also operate in mCherry-d.

We also note an interesting large shift in the I-ring dihedral, which fluctuates from −40° to 40° and is centered at approximately −20° in mCherry-d. We attribute this to these mutations, K70R and I197R, which alter the chromophore cavity such that the α-helix core and the chromophore cavity are destabilized. I-twisting is associated with nonradiative decay for HBDI, but in an FP, the I-ring is bound on either side to the protein matrix.^41,42^ This suggests the possibility that the observed dark state is associated with a twisted I-ring and thus photodestructive but potentially stable as it cannot complete isomerization due to chromophore–protein interactions. Similarly twisted structures, albeit with a protonated chromophore, have been shown to participate in dark states in other FPs.^43^ However, more work is needed to characterize this possibility. On the other hand, the exact chemical nature of the photobleached state remains unclear and may vary among different FPs.^44^ Irreversible photobleaching originating from photodestructive dark states may proceed through formation of a chromophore dianion via photoreduction, or through oxygen-dependent pathways involving reactive oxygen species.^40,45^ In contrast, photoprotective dark states, formed primarily through chromophore photoisomerization, are proposed to escape irreversible photobleaching by entering a reversible photocycle, as described by the “circular restoration model”.^46^

In conclusion, the work presented here provides a theoretical framework to extract dark-state conversion rates from fluorescence measurements. We showed that both DSC and GSR contribute to the fast initial fluorescence decrease in mCherry-based RFPs and the amplitude of DSC is a robust parameter to quantify *τ*_dsc_. This method could easily be extended to other dyes or FPs that emit at different wavelengths, such as mGold2s, where a significant photoprotective dark state could possibly explain the robust photostability of the variant.^4^ Employing analytical derivation and numerical simulations, we explain the biexponential nature of photobleaching decays in these FPs and how it relates to the dark-state properties. By systematic photophysical investigations of mCherry and mCherry-d, we conclude that dark-state-mediated photobleaching occurs in such RFPs. Higher dark-state conversion could also explain other interesting advances in the field of microscopy, such as the larger magnetic field-dependent fluorescence change observed in mCherry-d compared to mCherry, where dark states such as triplets have been hypothesized to play a critical role.^47,48^ Intensity-dependent photobleaching measurements in these RFPs reveal that both the rate and the amplitude of such decay increased as the illumination intensity increased from 1 to 10 kW/cm^2^. Finally, with the help of molecular dynamics simulation, we observe a significant deviation of the chromophore I-ring dihedral angle from zero and its enhanced fluctuation, which might contribute to the dark-state behavior observed here. Collectively, the insights from this study will provide useful input for designing RFPs with better photostability and provide a foundation for engineering stable dark states, which is essential in many advanced localization-based super-resolution microscopies such as MINFLUX.^49^

## Supplementary Material





## Notes

Details of the photobleaching model, directed evolution of mCherry-d, measurement and analysis of photobleaching, ground state recovery, dark state conversion, and MD simulations (PDF)
Transparent Peer Review report available (PDF)
