# Supplementary material for: Dark-State-Mediated Photobleaching in mCherry-Based Red Fluorescent Proteins
Source: J Phys Chem Lett. 2026 Mar 16;17(12):3596–604. doi: 10.1021/acs.jpclett.5c04106 (PMC13034455; doi:10.1021/acs.jpclett.5c04106)
Supplement: Supplementary file 2 [file jz5c04106_si_002.pdf]

jz-2025-04106r.R1

Name: Peer Review Information for "Dark state-mediated photobleaching in mCherry-based red fluorescent proteins"

First Round of Reviewer Comments

Reviewer: 1

Comments to the Author
Manna et al. present a combined experimental and kinetic-modeling study of photobleaching in mCherry-derived red fluorescent proteins, focusing on a direct comparison between mCherry and the variant mCherry-d. The central message is that long-lived dark-state cycling can strongly modulate apparent photobleaching under continuous illumination, and that differences in dark-state branching and recovery kinetics between closely related proteins can account for distinct decay behaviors. The authors introduce a compact rate-equation framework to rationalize multi-exponential fluorescence decays and their dependence on excitation intensity, and they argue that tuning dark-state kinetics can effectively "protect" fluorophores from irreversible photobleaching. The work will be of particular interest to the quantitative fluorescence imaging and fluorescent protein engineering communities, and it is also practically actionable for microscopists because it implies simple strategies (tuning irradiance, frame rate, and illumination duty cycle (dark intervals)) to extend usable imaging time and reduce biases in intensity-based measurements.

This is a relevant and potentially impactful topic for J. Phys. Chem. Letters. The head-to-head comparison of two closely related fluorophores is a clear strength, and the attempt to connect macroscopic decay curves to microscopic kinetic parameters is appealing. The Supporting Information is substantial and contains important experimental constraints (notably a dedicated ground-state recovery measurement with variable dark time), which strengthens the overall study. That said, the mechanistic conclusions still rely heavily on kinetic fits, and the manuscript would benefit from clearer demonstrations of parameter robustness and from bringing the most decisive supporting evidence more prominently into the main text. In addition, the novelty would be clearer if the prior literature on mCherry dark states, multi-state brightness, and the decoupling between reversible dark-state conversion and irreversible photobleaching were cited more completely.

Major revisions

1.
A core claim is that differences in extracted kinetic parameters explain why mCherry-d bleaches differently from mCherry. However, multi-exponential decay curves measured across irradiance can often be reproduced by different combinations of rate constants

once a dark reservoir and an irreversible sink are included. The manuscript would be significantly strengthened by a minimal identifiability analysis, such as confidence intervals on fitted parameters (bootstrapping is sufficient), parameter correlations/covariance, or a sensitivity analysis indicating which parameters are robustly constrained by the data. This is important because the paper's mechanistic interpretation rests on comparing fitted rate constants between the two proteins.

2.
The Supporting Information includes a ground-state recovery (GSR) measurement using a pulsed excitation scheme with variable inter-pulse dark time, which is highly relevant for constraining reversible dark-state shelving versus irreversible loss. Since this point is central to the manuscript's interpretation, the authors should highlight these results more prominently in the main text (either by moving a key figure/panel or summarizing the main quantitative outcomes). In addition, the manuscript should explicitly connect the experimentally measured recovery behavior to the parameters used in the continuous-illumination photobleaching model. If comparable GSR measurements exist for both mCherry and mCherry-d, presenting a direct comparison would further strengthen the mechanistic argument.

3.
The rate-equation framework provides a useful phenomenological description, but some discussion points read as if fitted effective rates uniquely identify specific microscopic photochemical pathways. Given the limited number of independent observables, these assignments may not be unique. The authors should consistently frame such statements as "consistent with" or "suggestive of," and include a short clarification distinguishing directly measured quantities from model-inferred effective parameters.

Minor revisions

1.
Because intensity dependence is central to the analysis, the manuscript should clearly state the irradiance values at the sample plane, how they were calibrated, and briefly comment on whether any high-irradiance artifacts could influence apparent kinetics.

2.
Please specify whether fitting was performed globally or locally, what weighting was used, and provide representative residuals for at least one or two traces. This is a small addition that greatly increases confidence in the reported fits.

3.
Since the results have direct consequences for experimental design, a brief concluding statement outlining practical take-home guidance (reducing peak irradiance, introducing dark intervals, adjusting frame rate to allow recovery from dark states) would increase

accessibility and impact for the microscopy community without requiring additional experiments.

4.
To better position the present contribution within established literature, the authors should ensure they cite key prior work showing:

* long-lived dark states and intensity/pH-dependent flickering in mCherry and related monomeric RFPs (Hendrix et al. Dark states in monomeric red fluorescent proteins studied by fluorescence correlation and single molecule spectroscopy, Biophysical Journal 94 (2008) 4103-4113).

* the existence of multiple brightness states in mCherry in living cells (Wu et al. Distinctive fluorescence flicker and bleaching dynamics of mCherry and enhanced yellow fluorescent protein in living cells, Biophysical Journal 96 (2009) 2391-2404).

* mechanistic/structural evidence for distinct photobleaching regimes and dark-state-related pathways in fluorescent proteins, which is directly relevant to the present discussion (Duan et al. Structural Evidence for a Two-Regime Photobleaching Mechanism in a Reversibly Switchable Fluorescent Protein. J. Am. Chem. Soc. 135 (2013) 15841-15850).

* explicit experimental evidence that reversible dark-state conversion and irreversible photobleaching can be decoupled in mCherry-derived proteins, including the effect of CW versus pulsed illumination (Dean et al, Analysis of red-fluorescent proteins provides insight into dark-state conversion and photodegradation, Biophysical Journal 101 (2011) 961-969).

* broader mechanistic reviews on chromophore transformations and photophysics in red fluorescent proteins (Subach et al. Fluorescent proteins: turning on and turning off, Chemical Reviews 112 (2012) 142-171).

5.
Ensure all rate constants and symbols are defined at first use and used consistently throughout. Also clarify which rates are intrinsic versus intensity-dependent, so the reader does not have to infer this from context.

Recommendation
The study is of clear interest for J. Phys. Chem. Letters and is close to publishable. The Supporting Information provides key experimental constraints, but the main manuscript should better leverage these results and the kinetic interpretation would be strengthened by a minimal analysis of parameter robustness/identifiability. The manuscript would also benefit from a more complete positioning within the established literature on mCherry dark

states and the decoupling between reversible dark-state cycling and irreversible photobleaching. I therefore recommend revision.

Reviewer: 2

Comments to the Author
This manuscript "Dark state-mediated photobleaching in mCherry-based red fluorescent proteins" by P. Manna... R. Jimenez addressed a useful property in the fluorescent protein filed of broad interest, namely the non-fluorescent or dark state, using experimental and theoretical methods. The analytical solution of rate equations and MD simulations nicely demonstrate the difference between mCherry and mCherry-d with insights into the chromophore I-ring twist on the proposed photodestructive dark state. Minor revision is thus recommended before it can be further considered for publication at JPCL. The list of issues was provided to the authors as follows.

1. The illumination conditions should be briefly summarized since the relevant power densities and regimes vary a lot in the literature (lack of standardization), hindering a direct comparison.
2. The nature of "irreversible photobleaching" should be discussed, even though the understanding would be incomplete given the limitation of current study. For instance, the decomposition of chromophore by ejecting some moieties from the "D" state? Redox? Singlet or triplet oxygen species (the oxygen diffusion channel was mentioned later)? This point may be tied to the "photodestructive dark states" mentioned on page 9 versus the "photoprotective dark states".
3. In the same context, the significance of "high-irradiance conditions" can be clarified as the general readers may consider it a specialized topic (which lacks broad interest). If general principles have been deduced to potentially use photobleaching to benefit bioimaging applications, that should be mentioned (more than one sentence in the end).
4. "at different intensities" by the end of Fig. 2 caption should be better written as "under different irradiation intensities" to be clearer. In Fig. 2a, the FL, FB, and FR should be defined in the caption.
5. Some recent works on the P- and I-ring twists/H-bonding patterns with clear effects on FQY/brightness or dark states are relevant and can add to discussions particularly on the molecular origins of protein chromophore: Ref. 36 is incomplete/inaccurate as "2023" (J. Am. Chem. Soc. 2024, 146 (26), 17646–17658); Proc. Natl. Acad. Sci. U.S.A. 2025, 122 (32), e2508094122; J. Am. Chem. Soc. 2020, 142 (25), 10978–10988; Int. J. Mol. Sci. 2021, 22 (1), 445; to name a few representative ones.
6. The "exp" in Eq. 17-18 in the SI needs to better show the exponential (the exponent is currently detached and unclear). Also, the coefficient q in Eq. 11-12 in the SI should be q' ? Check those details as the readers may try to work out all the steps for modelling.

Author's Response to Peer Review Comments:

<u>Response to the reviewers, Manna et al.</u>

(The responses are shown in blue, the changes made in the manuscript is shown in green)

Reviewer(s)' Comments to Author:

Reviewer: 1

Recommendation: This paper is probably publishable, but major revision is needed; I do not need to see future revisions.

Comments: Manna et al. present a combined experimental and kinetic-modeling study of photobleaching in mCherry-derived red fluorescent proteins, focusing on a direct comparison between mCherry and the variant mCherry-d. The central message is that long-lived dark-state cycling can strongly modulate apparent photobleaching under continuous illumination, and that differences in dark-state branching and recovery kinetics between closely related proteins can account for distinct decay behaviors. The authors introduce a compact rate-equation framework to rationalize multi-exponential fluorescence decays and their dependence on excitation intensity, and they argue that tuning dark-state kinetics can effectively "protect" fluorophores from irreversible photobleaching. The work will be of particular interest to the quantitative fluorescence imaging and fluorescent protein engineering communities, and it is also practically actionable for microscopists because it implies simple strategies (tuning irradiance, frame rate, and illumination duty cycle (dark intervals)) to extend usable imaging time and reduce biases in intensity-based measurements.

This is a relevant and potentially impactful topic for J. Phys. Chem. Letters. The head-to-head comparison of two closely related fluorophores is a clear strength, and the attempt to connect macroscopic decay curves to microscopic kinetic parameters is appealing. The Supporting Information is substantial and contains important experimental constraints (notably a dedicated ground-state recovery measurement with variable dark time), which strengthens the overall study. That said, the mechanistic conclusions still rely heavily on kinetic fits, and the manuscript would benefit from clearer demonstrations of parameter robustness and from bringing the most decisive supporting evidence more prominently into the main text. In addition, the novelty would be clearer if the prior literature on mCherry dark states, multi-state brightness, and the decoupling between reversible dark-state conversion and irreversible photobleaching were cited more completely.

Response: We thank the reviewer for the positive feedback on our work. Below, we provide detailed responses to the points raised.

Major revisions

1. A core claim is that differences in extracted kinetic parameters explain why mCherry-d bleaches differently from mCherry. However, multi-exponential decay curves measured across irradiance can often be reproduced by different combinations of rate constants once a dark reservoir and an irreversible sink are included. The manuscript would be significantly strengthened by a minimal identifiability analysis, such as confidence intervals on fitted parameters (bootstrapping is sufficient), parameter correlations/covariance, or a sensitivity analysis indicating which parameters are robustly constrained by the data. This is important because the paper's mechanistic interpretation rests on comparing fitted rate constants between the two proteins.

Response: We thank the reviewer for this helpful comment. We agree that including confidence intervals for the fitted parameters strengthens the arguments presented in the manuscript. Accordingly, we have added the 95% confidence intervals for the ground-state recovery parameters (Table 1) and the photobleaching data (Table 2). For the ground-state recovery analysis, MATLAB's default covariance matrix method was used to estimate the confidence intervals. For the photobleaching data, bootstrapping analysis was performed to determine the confidence intervals. In addition, we have included the standard deviations from biological replicates (n = 4–5) for the DSC time constants in Table 1. The relatively narrow confidence intervals indicate that the fitted parameters are well constrained by the data.

2. The Supporting Information includes a ground-state recovery (GSR) measurement using a pulsed excitation scheme with variable inter-pulse dark time, which is highly relevant for constraining reversible dark-state shelving versus irreversible loss. Since this point is central to the manuscript's interpretation, the authors should highlight these results more prominently in the main text (either by moving a key figure/panel or summarizing the main quantitative outcomes). In addition, the manuscript should explicitly connect the experimentally measured recovery behavior to the parameters used in the continuous-illumination photobleaching model. If comparable GSR measurements exist for both mCherry and mCherry-d, presenting a direct comparison would further strengthen the mechanistic argument.

Response: We agree that including pulsed excitation data strengthens the mechanistic argument for dark state-mediated photobleaching. Accordingly, we compared the photobleaching decays of mCherry and mCherry-d under continuous and pulsed excitation (Figure 3, main text). We have added a paragraph in the main text to explain these observations and included a detailed description of the experimental procedure and analysis in the Supporting Information (Section S10).

Under pulsed excitation, mCherry and mCherry-d photobleach approximately threefold and fivefold more slowly, respectively, than under continuous illumination. We attribute this behavior to the fact that pulsed excitation facilitates depopulation of photodestructive dark states during the inter pulse intervals, thereby improving photostability. These results further support the presence of photodestructive dark states in these fluorescent proteins.

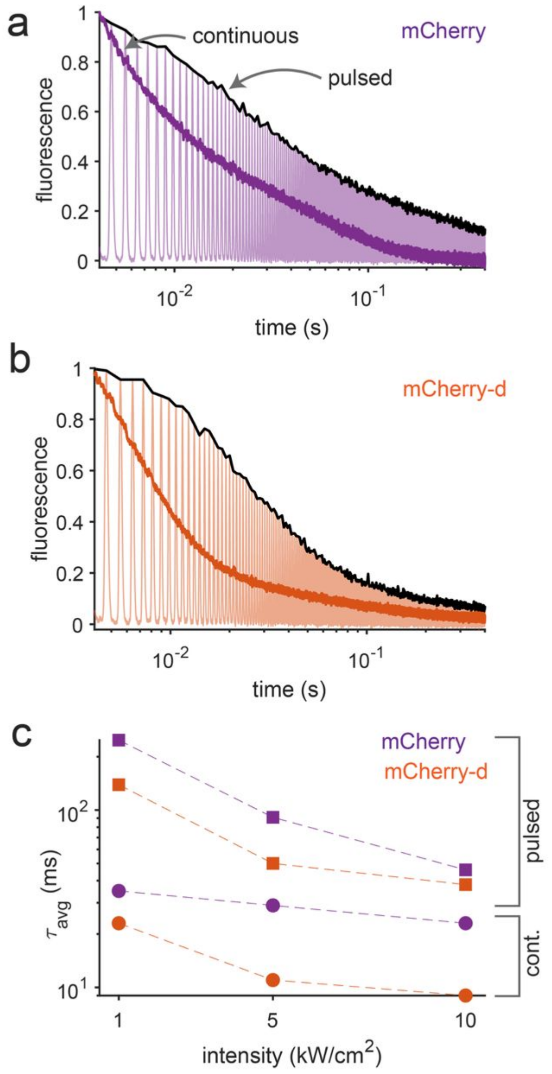

Figure 3: **Photobleaching decays under pulsed illumination**. Representative photobleaching decays at an average illumination intensity of 5 kW/cm^2 (measured at the sample plane) for mCherry (a) and mCherry-d (b). The normalized continuous photobleaching decays are shown in dark purple and dark orange for mCherry and mCherry-d, respectively. The normalized fluorescence obtained under pulsed illumination conditions is shown in light purple and light orange for mCherry and mCherry-d, respectively. In the pulsed mode, a Gaussian-shaped excitation beam with a FWHM of 0.2 ms and inter pulse delay of 0.65 ms was used. The peaks of the fluorescence measured under this condition are connected to generate the pulsed photobleaching decays (black lines). (c) Average photobleaching decays of mCherry (purple) and mCherry-d (orange) under continuous (filled circles) and pulsed (filled squares) illumination conditions.

We note that the temporal profile of the pulsed excitation used in the photobleaching experiments differs from that used for the ground-state recovery measurements. For photobleaching, the excitation pulse had a Gaussian profile with a full width at half-maximum (FWHM) of 0.2 ms and an inter pulse delay of 0.65 ms. This pulse scheme was chosen to mimic the illumination conditions experienced by cells expressing fluorescent proteins in the microfluidic sorter.

3. The rate-equation framework provides a useful phenomenological description, but some discussion points read as if fitted effective rates uniquely identify specific microscopic photochemical pathways. Given the limited number of independent observables, these assignments may not be unique. The authors should consistently frame such statements as "consistent with" or "suggestive of," and include a short clarification distinguishing directly measured quantities from model-inferred effective parameters.

Response: We agree that the observed data can be explained by more than one photophysical model. Accordingly, we have revised the manuscript to clarify this point and to explicitly distinguish between directly measured parameters and those inferred from the model, wherever applicable.

Minor revisions

1. Because intensity dependence is central to the analysis, the manuscript should clearly state the irradiance values at the sample plane, how they were calibrated, and briefly comment on whether any high-irradiance artifacts could influence apparent kinetics.

Response: We have clarified in the revised manuscript that all illumination intensities were measured at the sample plane. In addition, we have included a brief paragraph in the Supporting Information describing the procedure used to determine the irradiance. The relevant added text (shown in green) is provided below:

"The details of the ground state recovery measurements can be found here.[13] Briefly, the yeast cells expressing mCherry-d was irradiated with a 532 nm laser pulse sequence at 10 kW/cm^2 (intensity measured at the sample plane) similar to shown in Figure S7 a.

The excitation intensity at the sample plane was determined using a power meter to measure the laser power (P). The intensity (I) was calculated as:

$$I = \frac{P}{a}$$

where a is the area of the laser spot at the sample plane.

To determine the laser spot area, a fluorescent sample was imaged under low illumination intensity to avoid detector saturation. The diameter of the illuminated spot was measured in pixels using

ImageJ. The pixel-to-micrometer conversion factor was obtained by imaging a calibrated stage micrometer (standard grid), allowing conversion of the measured diameter from pixels to micrometers. The spot area was then calculated assuming a circular beam profile."

At sufficiently high excitation intensities, the excited-state population may access higher-lying excited states. In this regime, the kinetic model described in Figure 1 (main text) and the corresponding equations may not adequately capture the underlying photophysics.

2. Please specify whether fitting was performed globally or locally, what weighting was used, and provide representative residuals for at least one or two traces. This is a small addition that greatly increases confidence in the reported fits.

Response: Dark-state conversion (DSC), ground-state recovery (GSR), and pulsed photobleaching decays were fit locally. For the DSC decays, higher weights (1000:1) were assigned to the 0–1 ms time regime to accurately capture the DSC amplitude. In contrast, photobleaching decays under continuous illumination were fit globally to a biexponential function, where τ_1 was treated as a shared parameter across datasets and a_1 , a_2, τ_2 were allowed to vary with excitation intensity.

These fitting procedures are now clarified in both the main text and the Supporting Information. In addition, we have included the residuals for the DSC and photobleaching fits (Figures S8–S10) to demonstrate the quality of the fits.

3. Since the results have direct consequences for experimental design, a brief concluding statement outlining practical take-home guidance (reducing peak irradiance, introducing dark intervals, adjusting frame rate to allow recovery from dark states) would increase accessibility and impact for the microscopy community without requiring additional experiments.

Response: We thank the reviewer for this suggestion. We have added a few lines providing practical, take-home guidance based on our observations. The relevant text is as follows:

"Nevertheless, the comparative photobleaching studies presented above indicate that FPs possessing photodestructive dark states, such as mCherry, exhibit improved photostability under pulsed illumination. Additionally, lower excitation intensities and FPs with faster ground state recovery reduce population trapping in dark states, thereby mitigating irreversible photobleaching.[36]"

4. To better position the present contribution within established literature, the authors should ensure they cite key prior work showing:

* long-lived dark states and intensity/pH-dependent flickering in mCherry and related monomeric RFPs (Hendrix et al. Dark states in monomeric red fluorescent proteins

studied by fluorescence correlation and single molecule spectroscopy, Biophysical Journal 94 (2008) 4103-4113).

* the existence of multiple brightness states in mCherry in living cells (Wu et al. Distinctive fluorescence flicker and bleaching dynamics of mCherry and enhanced yellow fluorescent protein in living cells, Biophysical Journal 96 (2009) 2391-2404).

* mechanistic/structural evidence for distinct photobleaching regimes and dark-state-related pathways in fluorescent proteins, which is directly relevant to the present discussion (Duan et al. Structural Evidence for a Two-Regime Photobleaching Mechanism in a Reversibly Switchable Fluorescent Protein. J. Am. Chem. Soc. 135 (2013) 15841-15850).

* explicit experimental evidence that reversible dark-state conversion and irreversible photobleaching can be decoupled in mCherry-derived proteins, including the effect of CW versus pulsed illumination (Dean et al, Analysis of red-fluorescent proteins provides insight into dark-state conversion and photodegradation, Biophysical Journal 101 (2011) 961-969).

* broader mechanistic reviews on chromophore transformations and photophysics in red fluorescent proteins (Subach et al., Fluorescent proteins: turning on and turning off, Chemical Reviews 112 (2012) 142-171).

Response: The suggested papers are cited to the relevant part of the manuscript.

4. Ensure all rate constants and symbols are defined at first use and used consistently throughout. Also, clarify which rates are intrinsic versus intensity-dependent, so the reader does not have to infer this from context.

Response: We have ensured that all rate constants and symbols are defined at first use and used consistently throughout. We have clarified the intrinsic and intensity-dependent rates in the manuscript. Here is the relevant part added (shown in green) to the main text:

“Moreover, the DSC amplitude increases from 0.23 in mCherry to 0.64 in mCherry-d, indicating a higher propensity of mCherry-d to populate dark states. In our model, although the amplitude of DSC (a_{dsc}) is intensity-dependent (Eqn. 2), the corresponding time constant (τ_{dsc}) is treated as an intrinsic property of the fluorescent proteins. Similarly, τ_{gsr} was modeled as an intrinsic property of the sample. Consistent with this assumption, measurements of GSR time constants for RFPs acquired at different excitation intensities did not show any significant variation (Manna et al., 2015).”

Also here:

“Therefore, for each sample, decays with different intensities are globally fit with a bi-exponential function where τ_1 is kept as a shared variable. On the other hand, the amplitudes (a_1, a_2) and τ_2

are kept as intensity data dependent. The fits are shown as black lines in Figure 2e. Here, τ_1 is the reciprocal of k_{S1B} and therefore is an intrinsic time-constant whereas τ_2 is an intensity-dependent parameter."

Recommendation

The study is of clear interest for J. Phys. Chem. Letters and is close to publishable. The Supporting Information provides key experimental constraints, but the main manuscript should better leverage these results and the kinetic interpretation would be strengthened by a minimal analysis of parameter robustness/identifiability. The manuscript would also benefit from a more complete positioning within the established literature on mCherry dark states and the decoupling between reversible dark-state cycling and irreversible photobleaching. I therefore recommend revision.

Reviewer: 2

Recommendation: This paper is publishable subject to minor revisions noted. Further review is not needed.

Comments: This manuscript, "Dark state-mediated photobleaching in mCherry-based red fluorescent proteins" by P. Manna… R. Jimenez, addressed a useful property in the fluorescent protein field of broad interest, namely the non-fluorescent or dark state, using experimental and theoretical methods. The analytical solution of rate equations and MD simulations nicely demonstrates the difference between mCherry and mCherry-d, with insights into the chromophore I-ring twist on the proposed photodestructive dark state. Minor revision is thus recommended before it can be further considered for publication at JPCL. The list of issues was provided to the authors as follows.

Response: We thank the reviewer for the positive comments about our work. Below, we address the points raised by the reviewer.

1. The illumination conditions should be briefly summarized since the relevant power densities and regimes vary a lot in the literature (lack of standardization), hindering a direct comparison.

Response: We agree that information on illumination intensities help in direct comparison. We have added the relevant information in the main text, supporting information and also in the Figure captions. Here is a relevant portion of the manuscript that reflects the change (shown in green):

A 532 nm laser, operating at an intensity of 10 kW/cm^2 (measured at the sample plane), was used to illuminate the cells expressing RFPs. Additional details of the GSR measurements and analysis are provided in SI Sec S7.

We next focus on the dark state conversion (DSC) kinetics. For the measurements of DSC time-constants, RFPs expressed in yeast cells were irradiated with a 561 nm laser at 5 kW/cm^2 in a similar set-up to that in. [25]

2. The nature of "irreversible photobleaching" should be discussed, even though the understanding would be incomplete given the limitations of the current study. For instance, the decomposition of chromophore by ejecting some moieties from the "D" state? Redox? Singlet or triplet oxygen species (the oxygen diffusion channel was mentioned later)? This point may be tied to the "photodestructive dark states" mentioned on page 9 versus the "photoprotective dark states".

Response: Based on the published works, we think irreversible photobleaching in FPs with photodestructive dark states (mCherry, mFruits) proceed through formation of chromophore dianion radical or through oxygen-dependent pathways. On the other hand, in photoprotective dark states (FusionRed, TagRFP-T) those are formed by chromophore cis-trans isomerization "short circuit" the irreversible photobleaching by entering a reversible photocycle. We have added this discussion in the main text (shown in green):

Similarly twisted structures, albeit with a protonated chromophore, have been shown to participate in dark states in other FPs.[43] However, more work is needed to further characterize this possibility. On the other hand, the exact chemical nature of the photobleached state remains unclear and may vary among different FPs.[44] Irreversible photobleaching originating from photodestructive dark states may proceed through formation of a chromophore dianion via photoreduction, or through oxygen-dependent pathways involving reactive oxygen species.[40,45] In contrast, photoprotective dark states—formed primarily through chromophore photoisomerization—are proposed to escape irreversible photobleaching by entering a reversible photocycle, as described by the "circular restoration model."[46]

3. In the same context, the significance of "high-irradiance conditions" can be clarified as the general readers may consider it a specialized topic (which lacks broad interest). If general principles have been deduced to potentially use photobleaching to benefit bioimaging applications, that should be mentioned (more than one sentence in the end).

Response: We thank the reviewer for the suggestion. The intensity used in this work (1-10 kW/cm^2) is similar to what is used in laser scanning confocal microscopy. This has been clarified in the text.

mCherry and mCherry-d are closely related and have similar molecular brightness (*i.e.*, extinction coefficient × fluorescence quantum yield) yet display contrasting dark state and photobleaching properties upon illumination in kW/cm^2 regimes (1-10 kW/cm^2). This intensity regime is relevant for typical confocal laser scanning microscopies.

4. “at different intensities” by the end of Fig. 2 caption should be better written as “under different irradiation intensities” to be clearer. In Fig. 2a, the FL, FB, and FR should be defined in the caption.

Response: We have changed the captions as per the suggestion. Here is the relevant portion of the caption after this modification. The change is shown in green.

“Values of FL, FB, and FR are extracted from these experiments to calculate percent recovery employing Eqn. 4. Here, FL and FR represent the fluorescent intensities at the beginning of the first and second pulses, respectively, whereas FB denotes the fluorescence intensity measured at the end of the first pulse.”

“Normalized photobleaching decays of mCherry-d at 1, 5, and 10 kW/cm^2. For each sample, the decays at different irradiation intensities are globally fit with a bi-exponential function (black lines).”

5. Some recent works on the P- and I-ring twists/H-bonding patterns with clear effects on FQY/brightness or dark states are relevant and can add to discussions particularly on the molecular origins of protein chromophore: Ref. 36 is incomplete/inaccurate as “2023” (J. Am. Chem. Soc. 2024, 146 (26), 17646–17658); Proc. Natl. Acad. Sci. U.S.A. 2025, 122 (32), e2508094122; J. Am. Chem. Soc. 2020, 142 (25), 10978–10988; Int. J. Mol. Sci. 2021, 22 (1), 445; to name a few representative ones.

Response: Thanks for pointing out the incomplete Ref 36. We have added the completed references and also added the references suggested by the reviewer in the relevant part of the manuscript.

6. The “exp” in Eq. 17-18 in the SI needs to better show the exponential (the exponent is currently detached and unclear). Also, the coefficient q in Eq. 11-12 in the SI should be q’? Check those details, as the readers may try to work out all the steps for modelling.

Response: Thanks for pointing this out. We have corrected Eq. 17-18 and Eq. 11-12 in the SI. Also, we have checked all the variables carefully to make sure everything is consistent.
